# Soldiers, not by choice: confronting conscription as a public health harm

**DOI:** 10.1016/j.lanepe.2025.101461

**Published:** 2025-09-08

**Authors:** Samuel Christopher Rogers, Ralph Hurley O'Dwyer

**Affiliations:** a School of Religion, Theology and Peace Studies, Trinity College Dublin, Ireland; b The Fletcher School of Law and Diplomacy, Tufts University, Medford, MA, USA; c Department of Public Health, HSE Dublin and Midlands, Dr Steevens' Hospital, Dublin 8, Ireland

We read with interest the editorial “Soldiers of Ukraine” and echo the authors' concerns regarding the immense challenges facing Ukrainian soldiers, veterans, and their families since Russia's full-scale invasion in 2022.^1^ As the editorial rightly notes, these harms extend far beyond the battlefield. Ensuring that soldiers receive proper care is a moral and political imperative.

However, we would like to comment on the editorial's silence on the matter of military conscription. While we condemn Russian aggression and acknowledge the national emergency in Ukraine precipitated by Russia's invasion, we argue that conscription still warrants critical scrutiny as a bioethical and public health concern. Since martial law was declared in 2022, the Ukrainian government has prevented male citizens aged 18–60 (with a few exceptions) from leaving Ukraine. While many men have voluntarily joined the armed forces, thousands of others have been conscripted and subject to non-voluntary military deployment.

We propose that conscription is not an ethically neutral component of war, but a public health crisis in its own right.^2^ By eliminating the moral agency and bodily autonomy of individual citizens, conscription amounts to a form of structural violence^3^ and compounds burdens already borne by volunteer soldiers. One such burden is *moral injury*, the distress of being forced to act against one's ethics. First conceptualised in relation to American conscripts during the Vietnam War, it is strongly associated with PTSD, depression, and substance misuse.^4^

Although comprehensive health data on Ukrainian conscripts is limited due to the ongoing nature of the conflict, emerging studies are capturing the war's varied mental health implications.^5^ Research from comparable contexts link compulsory military service with long-lasting physical and psychological harm. A study of Turkish conscripts found that those exposed to conflict zones were more likely to experience depressive symptoms, even decades after discharge.^6^ Even in the absence of active combat, conscription carries harm. For example, a study of conscripts in South Korea associates compulsory military service with long-term increases in tobacco and alcohol use.^7^

Beyond the individual, the harms of conscription extend to wider society. As a social determinant of health, it contributes to long-term illness—straining healthcare systems—and also removes young, healthy individuals from the workforce—undermining both economic and public health resilience. Lastly, by separating children from fathers, it may compound the already significant psychosocial effects of war on children in Ukraine.^8^

Acknowledging the unjust and brutal nature of the Russian invasion does not require the suspension of ethical or health-based scrutiny for state policies, including conscription. Instead, the extreme nature of the context heightens the importance of safeguarding Ukrainian citizens’ psychological and physical well-being amidst this time of immense uncertainty.

Its documented harms challenge any assumption that conscription is ethically neutral. As a state policy, it violates the four most commonly recognised principles of contemporary medical ethics: autonomy, non-maleficence, beneficence, and justice.^2^ Yet conscription seems to occupy a unique space in commentary regarding modern conflict—overlooked as incidental or inevitable and left out of most conversations regarding the health consequences of war.

We support the editorial's call for integrated mental health care, veteran support services, and international investment in post-war recovery in Ukraine. However, these measures must include a reckoning with the health consequences of military conscription. In amplifying the editorial's call for comprehensive care and justice for Ukraine's soldiers, we urge the global health community to also confront the ethical and public health implications of compulsory military service, as a harm necessitating consideration in its own right. This reckoning is not only a moral and political imperative, but also crucial to laying the groundwork for a healthier, more resilient post-war Ukraine.

## Contributors

Samuel Christopher Rogers: conceptualisation, writing–original draft, writing–review and editing; Ralph Hurley O'Dwyer: conceptualisation, writing–original draft, writing–review and editing.

## Declaration of interests

The authors declare no competing interests.
